# Methods for the In Vitro Characterization of Nanomedicines—Biological Component Interaction

**DOI:** 10.3390/jpm7010002

**Published:** 2017-01-27

**Authors:** Cristina Fornaguera, Conxita Solans

**Affiliations:** 1 Sagetis-Biotech, Barcelona, 08017, Spain; 2 Institute of Advanced Chemistry of Catalonia (IQAC-CSIC) and Networking Centre on Bioengineering, Biomaterials and Nanomedicine (CIBER-BBN), Barcelona, 08034, Spain; conxita.solans@iqac.csic.es

**Keywords:** Personalized nanomedicine, characterization techniques, nanomaterials, interaction with biological components

## Abstract

The design of colloidal nanosystems intended for biomedical applications, specifically in the field of personalized medicine, has increased notably in the last years. Consequently, a variety of characterization techniques devoted to studying nanomedicine interactions with proteins and cells have been developed, since a deep characterization of nanosystems is required before starting preclinical and clinical studies. In this context, this review aims to summarize the main techniques used to assess the interaction of nanomedicines with biological systems, highlighting their advantages and disadvantages. Testing designed nanomaterials with these techniques is required in order to have more information about their behavior on a physiological environment. Moreover, techniques used to study the interaction of nanomedicines with proteins, such as albumin and fibrinogen, are summarized. These interactions are not desired, since they usually are the first signal to the body for the activation of the immune system, which leads to the clearance of the exogenous components. On the other hand, techniques for studying the cell toxicity of nanosystems are also summarized, since this information is required before starting preclinical steps. The translation of knowledge from novel designed nanosystems at a research laboratory scale to real human therapies is usually a limiting or even a final point due to the lack of systematic studies regarding these two aspects: nanoparticle interaction with biological components and nanoparticle cytotoxicity. In conclusion, this review will be a useful support for those scientists aiming to develop nanosystems for nanomedicine purposes.

## 1. Introduction

**Figure 1:**
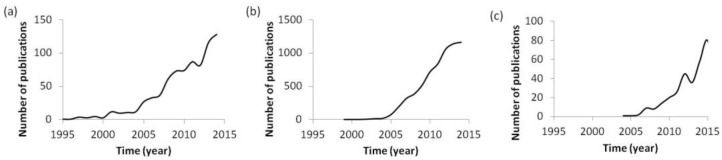
Number of publications per year, indexed in Scopus that contain the term (**a**) “nanosystem,” (**b**) “nanomedicine,” and (**c**) “personalized nanomedicine.”

Nanosystems and nanomaterials are general terms to designate any entity with at least one dimension having sizes ranging in the nanometric scale, which includes a substantial variety of nanoentities with different properties, such as nano-emulsions, nanoparticles, polyplexes, dendritic structures, micelles, and liposomes, among others [1,2,3]. Interest in nanosystems has experienced an exponential increase since the first reports appeared in the nineties (Figure 1a). Specifically, the interest in their use for biomedical applications, a field in which they are called nanomedicines, has increased notably during the last 20 years (Figure 1b) [4,5,6,7] due to the fact that current treatments have not yet solved certain drawbacks, such as the controlled release of therapeutic compounds or their biodistribution. In parallel, there has been an exponential increase in the impact of personalized medicine on nanotherapies (Figure 1c). Since the beginning of gene therapy, personalized medicine has arisen as an opportunity to treat each individual according to the specific requirements defined in their genome; so individual requirements must be taken into account when designing nanosystems. It is thought that, by combining the advantages of both fields, nanotechnology and personalized medicine, very efficacious treatments will be developed in the next few years.

In conventional treatments, high doses of actives are required, which usually produce severe side effects. In some cases (e.g., genetic diseases affecting different genes such as cystic fibrosis or heterogeneous gene expression in cancers), the treatment proposed is not effective for all patients. In this context, novel delivery nanosystems are advantageous for personalized therapies in that 1) they protect the actives they encapsulate; 2) they enable the controlled and sustained release of actives, allowing a decrease in therapeutic concentrations; 3) they can be tuned to reach their targeting organs, thus enabling a localized active release and a reduction in side effects; and 4) their surface can be tailored with a variety of chemical moieties to achieve multifunctional therapeutics specific to each individual [4,6,7,8,9,10,11]. The numerous advantages that nanomedicines possess have been perceived by the pharmaceutical industry, thus resulting in the industrial production and commercialization of a variety of nanomedicines, such as the widely known Abraxane® (Celgene corporation, Berkeley Heights, N.J., United States), a nanomedicine approved in 2005 due to its proven efficacy in various cancers, which consist in a nanoparticle dispersion loaded with paclitaxel [6,7,12,13,14]. It is also remarkable that, not only the therapeutic applications of nanomedicines are of interest, but also their applications as diagnostic nanosystems (e.g., polymeric nanoparticles labeled with radioactive isotopes to image tumors or Lumirem^®^^®^ (AMAG Pharmaceuticals Inc., Waltham, MA, United States) and Endorem^®^ (AMAG Pharmaceuticals Inc.), iron oxide nanoparticles as contrast agents to image the gastrointestinal tract), and even for theranostic purposes, that is, the combination of therapy and diagnosis (e.g., crosslinked dextran iron oxide nanoparticles loaded with near-infrared (NIR) fluorophores for the photodynamic therapy in artherosclerotic lesions) [6,14,15].

**Figure 2:**
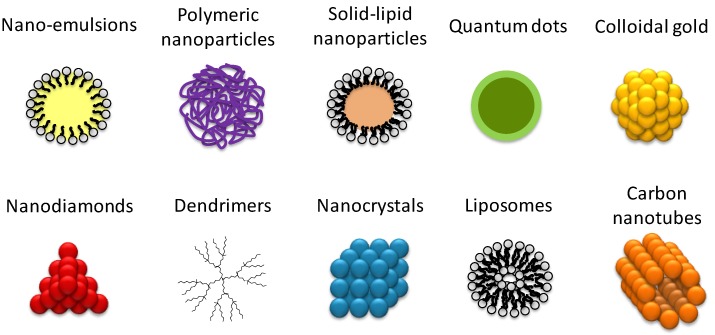
Schematic representation of the most commonly used nanomedicine types composed of different kinds of materials.

Prior to the transference of colloidal nanosystems to pharmaceutical industries and their commercialization, and after characterization of their physico-chemical properties, a deep study of their interaction with biological components is required to ensure that safe nanomedicines are obtained and that their behavior in physicological conditions is clearly assessed. Currently, agencies such as the Organisation for Economic Co-operation and Development (OECD) have already published guidelines for evaluating the safety of manufactured nanomaterials, and reviews with the objective of summarizing the state of the art of nanosafety have appeared [15]. Nevertheless, although substantial efforts have been made in the field of nanomedicine characterization [6,16], for example, by the Nanotechnology Characterization Laboratory in Frederick, MD, USA, nowadays, standardized protocols, assays, and/or methodologies for an adequate characterization of colloidal nanomaterials before preclinical assays do not yet exist, and the Food and Drug Administration (FDA) has not yet formulated guidelines [3,12,16]. However, several reviews have appeared with the objective of summarizing characterization techniques for nanomaterials, mainly for nanoparticles [2,3,6,16,17,18]. Each of them gives a particular point of view of the most relevant techniques in studying nanomaterials. Most of them point out the importance of characterizing nanomedicines in physiological conditions to avoid misleading results, since environmental conditions affect nanomaterial properties [2,17,18], but the interactions of nanomaterials with biological components have only been described in a few of them, and one brief description, without specifying useful techniques, has been given [17,18]. In addition, most of these reviews focus mainly on nanoparticles [2,3,6,17,18]. It is a fact that the available information is dispersed in the current literature, and a single review taking into consideration novel nanomaterial development is missing. The objective of this work is to review currently available techniques for a complete characterization of nanomaterial interaction with biological components, which must be addressed when designing nanomedicines before starting clinical and preclinical studies. For each parameter studied, different techniques are described, highlighting their advantages and disadvantages, indicating which nanomaterials can be properly studied by each technique. Hereafter, the terms nanosystems, nanomedicines, and colloidal nanomaterials will be used as general synonyms to name nanostructures with nanometric dimensions, independently of the specific entity they represent (e.g., nanorods or nanoparticles), their composition (e.g., polymer or metal), and their use (e.g., drug delivery systems or non-viral gene delivery systems) (Figure 2).

## 2. Experimental Techniques for the Analysis of Nanoparticle Interaction with Biological Components

A deep physicochemical characterization of designed nanomaterials, intended for biomedical applications, is required before in vitro efficacy studies [3]. The complexity involved in the characterization of nanosystems is noteworthy, as they are usually composed of various parts that must be fully characterized individually and together in the nanomedicine (e.g., multifunctional nanoparticles with encapsulated compounds and various functionalization moieties) [6]. In general, key parameters to study can be classified in three types of techniques: analytical, colloidal, and biological [2,3,12,17,19]. In this review, a deep description of the third type of technique is detailed—those aimed to define the interaction of nanomaterials with biological components in a physiological environment.

### 2.1. Techniques to Study Interaction of Nanomaterials with Proteins

Most therapeutic nanosystems are designed for intravenous systemic administration (the most restrictive parenteral administration route), targeting the desired organ. Therefore, prior to reaching the target tissue, they come into contact with many proteins in the blood. For this reason, the characterization of nanomaterials’ interaction with proteins is of utmost importance, since they must be specifically designed to avoid non-desired interaction with any kind of protein. Techniques to study these interactions are specified below. In brief, the stability of nanomaterials in a physiological environment is firstly described. Electrophoretic techniques for studying the interaction of nanomaterials with proteins are described (mixture of different kinds of proteins or a specific individual protein), and a specific case of interactions with blood components is also discussed. 

#### 2.1.1. Nanomaterial Stability in a Physiological Environment

Prior to studying a specific protein interaction, it is important to confirm nanomaterial stability in a biological environment, simulating physiological conditions, such as the widely known fetal bovine serum (FBS) protein solution. Therefore, the preliminary study of nanomaterial interactions with proteins can be performed without the need of any biological system, using only the purified proteins. Many techniques, most of which are summarized in a previous study of Fornaguera et al., [20], and which have been used to assess the stability of nanosystems in physiological conditions, determine the stability of poly-(D,L-lactide-co-glycolic acid) PLGA nanoparticles with different modifications in a protein solution. In general, these techniques are recommended as a first step in the characterization of nanomaterial–biological component interactions because, since they do not use biological material (except for the purified proteins solutions), they are less costly, safer, faster, and easier to perform than in vitro and in vivo studies.

Firstly, it is recommended that a key parameter of the nanomaterial, such as droplet size and/or surface charge, as a function of time, is measured under incubation at 37 ºC with FBS, since this is the temperature of physiological systems and FBS is representative of the whole blood protein solution. If changes in the measured parameter take place, it is also recommended that individual proteins are deeply studied to find out which one is producing the change (i.e. the one interacting with the nanomaterial). It should be noted that the study of interactions with serum of different origins can result in divergent results. Serum is a biological component, obtained from animals or individuals. Therefore, the reproducibility of the results can be difficult when changing the serum origin because of the inter-individual variability that could result in the identification of different interactions. For this reason, the testing of serums from different species (e.g., human and bovine) and from different commercial brands is strongly recommended, since the results can give a complete view of the expected in vivo behavior of designed nanomaterials in a protein environment.

**Figure 3:**
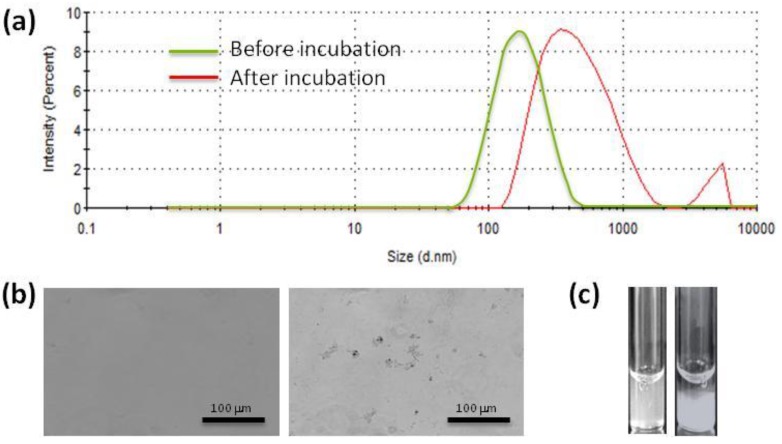
(**a**) Hydrodynamic size distribution. (**b**) Optical micrography. (**c**) Macroscopic appearance of a poly-(D,L_lactide-co-glycolic acid) PLGA nanoparticle dispersion before (left) and after (right) being incubated with fibrinogen.

Concerning individual proteins study, albumin is the most abundant protein in the blood, so it is the first candidate to test. In parallel, fibrinogen, among others, can also be tested, since it represents a different group of proteins due to its elongated shape, as compared to the spherical 3D conformation of albumin [10,20]. When incubating nanoparticles with albumin or fibrinogen, the droplet size of nanoparticles can notably increase due to the formation of microscopic aggregates, which thus does not enable measurement by light scattering techniques (Figure 3a). However, the presence of aggregates can be confirmed under optical microscopy (see Figure 3b as an example). If the aggregation is substantial, macroscopic sediment will also appear (Figure 3c). 

It is worth noting that physiological conditions may vary in diseased individuals, as compared to healthy individuals (e.g., increase on the body temperature, decrease on blood metabolites such as insulin). For this reason, to design a personalized therapy for each patient, physiological conditions to test the stability of nanomaterials must be tuned in order to simulate the environment of each patient. However, achieving commercial protein mixtures that simulate disease conditions can be much more difficult than using healthy materials, which could complicate the studies and even result in laboratory results that are not translatable to physiological conditions. In this case, after the laboratory study of the interactions with conditions similar to those of disease individuals, it is recommended that protein interactions in an animal model of the disease are studied to find out if the expected behavior of the designed nanomaterial takes place.

#### 2.1.2. Electrophoretic Techniques Used to Characterize Nanomaterial–Protein Interactions

Electrophoretic techniques (summarized in Table 1) are also used at the nanoscale to study the interaction between nanomaterials and proteins. These techniques consist in the migration of charged molecules (DNA, RNA, or proteins) through a polymeric matrix under an electric field provided by submerged electrodes, the migration being dependent on either the size or the isoelectric point of the studied material [21]. The polymeric matrix is usually composed of agarose to separate macromolecules such as nucleic acids, or of polyacrylamide to separate smaller molecules such as proteins [21]. They have been widely used in the nanomedicine field to determine hydrodynamic sizes and study the formation of protein corona on nanomaterial surfaces, and the adsorption of specific proteins onto nanomaterial surfaces [3,20,22,23,24]. Their main uses are specifically detailed below and summarized in Table 1. 

##### SDS-PAGE Electrophoresis

The sodium dodecyl sulfate–polyacrylamide gel electrophoresis (SDS-PAGE) is the most commonly used electrophoresis for studying proteins. It is a denaturing electrophoresis that, performed in a single dimension (the most common application), separates proteins as a function of their apparent molecular mass, since, after the denaturing process and incubation with SDS reagent, all proteins are equally negatively charged, without their 3D structure [21,25]. The size of the pores formed in the polymeric matrix depend on the percentage of polyacrylamide used [21].

**Figure 4:**
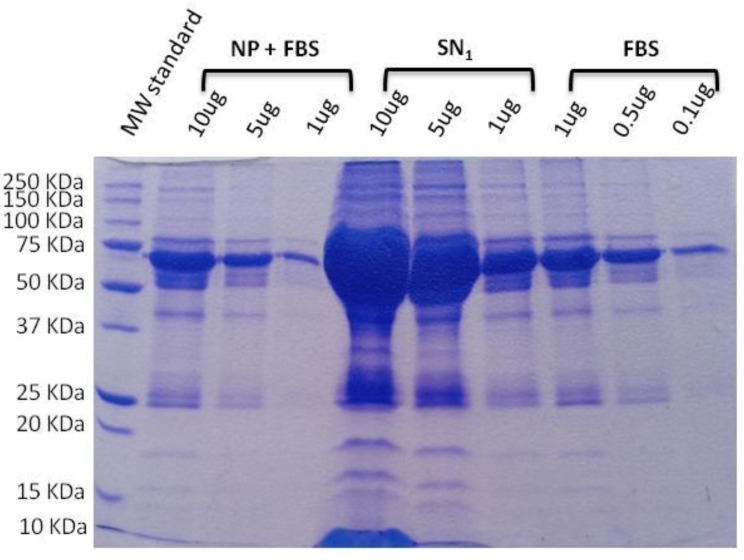
Example of a sodium dodecyl sulfate–polyacrylamide gel electrophoresis (SDS-PAGE) electrophoresis of poly-(D,L-lactide-co-glycolic acid) PLGA nanoparticles incubated with fetal bovine serum (FBS). Nanoparticle (NP) + FBS fraction correspond to those proteins strongly adsorbed onto nanoparticles surface, while the first supernatant SN1 fraction corresponds to non-adsorbed proteins. As it can be observed, the mass and amount of adsorbed proteins is lower than non-adsorbed [25].

SDS-PAGE electrophoresis is a simple and affordable technique that has been used prior to in vitro and in vivo studies to find out and quantify the proteins attached to nanoparticle surfaces, for example (see Figure 4) [21,26,27,28,29,30,35,36,37]. It is recommended as a first approximation that the level of protein corona formation on a nanomaterial surface, which is called opsonization and represents the first step in nanosystem phagocytosis, is known. It is not desired because phagocytic cells neutralize the nanomaterial and then cannot reach the target organ, thus incapable of performing the therapeutic action. When analyzing a library of formulations, this technique can be useful in selecting the one that is less opsonized. However, since it is a basic technique, the information that it gives is limited, and more specific techniques (detailed below) are recommended after this preliminary opsonization analysis. It has disadvantages, such as the number of preparative steps required prior to running the electrophoresis and the few semi-quantitative results that it yields [25].

##### Immunomethods

The terms immunomethods or immunochemical methods refer to a variety of immunoelectrophoretic methods, i.e., a variety of electrophoresis whose detection is performed through immunological techniques, using antibodies to detect the results [10]. Therefore, the high sensitivity of immunomethods is an advantage, in contrast to the high cost of using antibodies. Different kinds of agarose electrophoresis, where proteins migrate under various conditions, are performed. After the time left to run, proteins are detected through gel incubation with specific antibodies, which are further stained, for example, with Comassie blue. In this review, three kinds of immunomethods, which were considered of interest in the study of the interactions of proteins with nanomedicines, are described: rocket immunoelectrophoresis, radial immunodiffusion, and 2D-immunoelectrophoresis.

• Rocket immunoelectrophoresis

**Figure 5:**
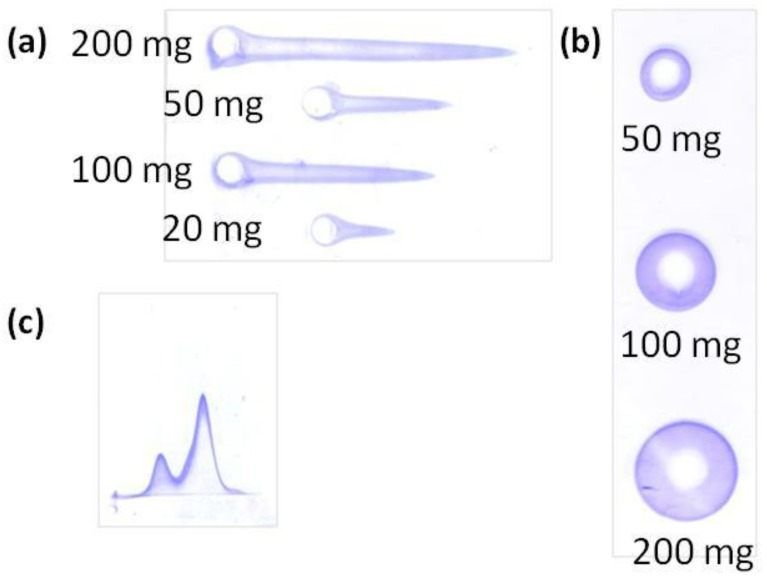
Examples of the gels obtained using described techniques. (**a**) Rocket immunoelectrophoresis of the free bovine serum albumin (BSA) after being incubated with polymeric nanoparticles at increasing concentrations. (**b**) Radial immunodiffusion of BSA after being incubated with polymeric nanoparticles at increasing concentrations. (**c**) 2D-immunoelectrophoresis of the C3 proteins after being incubated with polymeric nanoparticles (Adapted from [30]).

Rocket immunoelectrophoresis is a rapid, simple, and reproducible immunochemical technique to determine and quantify a single protein contained in a sample in which other components, even other proteins, can be present. Samples are loaded in circular wells at one edge of the agarose gels and migrate in the gel under an electric field. The protein migration results in a rocket-shape precipitation (Figure 5A), whose height is proportional to the protein concentration [10,29,30,36].

This method is widely used for the qualitative determination of albumin, usually bovine serum albumin (BSA), a protein that remains in solution after its incubation with different kinds of polymeric nanoparticles. However, it could be used for other types of nanosystems, since human serum albumin (HSA) is the major protein of the blood and its interaction with nanomaterials will influence their fate and biodistribution [10,37]. This method can also be used for the study of other kinds of proteins replacing albumin for the required protein. Nevertheless, since albumin is the main protein in blood, when studying other proteins, it must be taken into account that the behavior of individual minoritary isolated proteins could vary when majoritarian proteins are present. To circumvent this problem, a strategy to be applied is the incubation of nanomaterials with FBS or a protein mixture, albeit specifically detecting the desired protein with the appropriate monoclonal antibody.

• Radial immunodiffusion

Radial immunodiffusion is an immunochemical method for the quantification of a single protein contained in a sample with other components. Like the others, it consists in an agarose gel. In this method, however, the samples are loaded in circular wells at a central region of the gel and diffuse through the gel, forming a sedimentation ring (Figure 5b) whose diameter is proportional to the amount of free protein that has diffused [10]. 

As rocket immunoelectrophoresis, radial immunodiffusion has been widely used for the quantification of free BSA after its incubation with polymeric nanoparticles [10]. The difference between them is the concentration of proteins that they can detect: while rocket immunoelectrophoresis can detect protein concentrations as low as 5 μg/mL, radial immunodiffusion requires at least 50 μg/mL of proteins or higher to be detected. Therefore, it is recommended that both techniques are used in a study to have a wider concentration range.

Since both, radial immunodiffusion and rocket immunoelectrophoresis are similar, their advantages and drawbacks can be summarized together. An important advantage of both techniques is the ability to obtain quantitative results with little equipment and a rapid, easy, sensitive, and highly accurate method [10,24,31]. Another relevant advantage is that they can be performed directly with bioconjugates, without a separation step of proteins from nanomaterials, which is advantageous because separation steps could change the balance of attached/non-attached proteins and perturb the results. One drawback of these techniques is the requirement of running a calibration curve, necessary for the quantification of the studied proteins. The calibration curve is usually composed of known concentrations of the protein to test. For radial immunodiffusion, the calibration curve consists in a linear relationship between the square of the diameter of the sedimentation rings and the concentration of the protein in the well; for rocket immunoelectrophoresis, it is a linear relationship between the height of the immunoprecipitation peak and the protein concentration in the well [10,30]. Another important drawback is the difficulty of performing these techniques with surfactant-containing samples, since some surfactants, such as the widely used polysorbate 80, have been described to perturb the quantification of the results [38].

##### 2D-Immunoelectrophoresis to Detect Nanomaterial Interaction with C3 Complement Protein

2D immunoelectrophoresis is a very versatile technique that can be used for a multiplicity of purposes, all of which involve protein migration, such as the careful study of protein opsonization (using a non-denaturalizing gel): running the first dimension as a function of protein molecular weight and running the second dimension as a function of the isoelectric point (pI). Although many proteins are present in FBS, each spot of the 2D gel will represent a protein of a characteristic molecular weight and a characteristic pI, which can be attributed to a single protein. Since this technique has been used for a long time for many purposes, though its use in the nanomedicine field is limited, it is beyond the scope of the present review.

In this review, 2D immunoelectrophoresis is presented as a useful tool for studying the activation of the complement system, as an indication of the influence of nanomedicines on the immune system, which is a key parameter in studying nanomedicines before in vivo administration, since it will determine nanomedicine lifetime, fate, and biodistribution [32,33]. This technique specifically detects the interaction of the nanosystems with the C3 protein and entails the use of horizontal agarose protein electrophoresis in two dimensions. In the first dimension, the proteins are separated as a function of their molecular weight (the smaller the protein, the further it migrates), while they are separated in the second dimension as a function of their concentration (the higher the concentration, the further they run) (Figure 5C).

**Figure 6:**
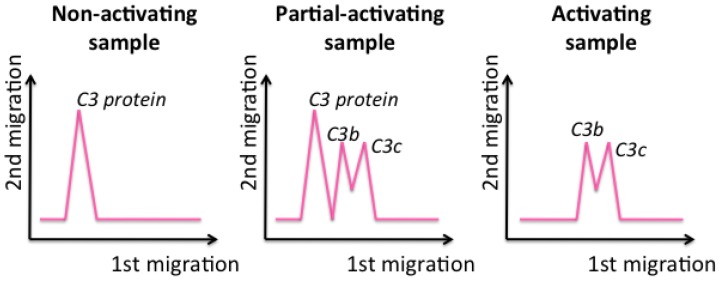
Schematic representation of 2D-immunoelectrophoresis gels when studying different types of samples.

To study the complement system activation, C3 protein is the best choice, since it is a key protein and the major protein of the complement cascade [32,33,39]. It has to be incubated with the samples to test. After incubation, samples are loaded into the well of the gel and migrate through the two dimensions. If the C3 protein is not activated, which means that nanomaterials do not influence the complement cascade, a single peak, at slow migrations in the first dimension, is obtained, corresponding to the entire C3 protein (Figure 6, left). In contrast, if a sample activates the C3 protein, it breaks into fragments that, since they are smaller, migrate longer in the first dimension and are detected as two peaks (Figure 6, right) [33].

This methodology is rapid, versatile, and easy; it can be applied with simple equipment. In addition, in a recent study, Coty et al. demonstrated the possibility of applying this technique to an analysis of up to 35 samples simultaneously, in a single electrophoresis run, which reduces the time and cost of this technique [34]. However, its sensitivity is low, and, if the nanosystems are composed of protein components, it will yield a higher interaction with the C3 protein, although this does not mean that a higher activation of the immune system is always produced (false positive) [20,32]. When designing nanomaterials for therapeutic applications, in many cases, activating the immune system is not desired, since, if it detects the administered nanosystems, it will activate the degradation of the component detected as exogenous. As a consequence, the nanosystems will be cleared, failing to enable their arrival to the target organ. 

Therefore, the application of this technique is recommended together with the study of protein corona formation (described above) for a preliminary study of the interaction of nanomaterials with proteins and their further detection and elimination by the immune system.

#### 2.1.3. Blood Coagulation Study

Coagulation or clotting time, which is related to the interaction of the nanosystems with proteins of the coagulation cascade, is another important parameter to be determined before preclinical studies. This interaction could lengthen or shorten the coagulation time; however, in general, it is not desired that the coagulation time be modified. Although standard values for the coagulation time exist, they can slightly vary for each individual. As stated earlier, these slight variations are especially important when designing personalized therapies, specifically in the case of diseased individuals, since some diseases can modify blood coagulation times (e.g., hemophilia), and controls of diseased blood are required to study the effect of nanomaterials.

**Figure 7:**
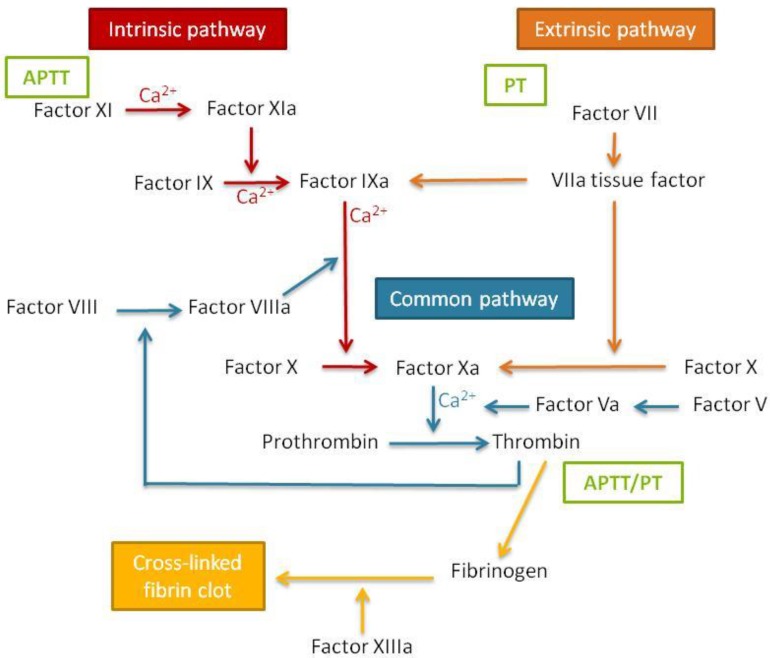
Schematic representation of the blood coagulation cascade. APTT: activated partial thromboplastin time; PT: prothrombin time.

Blood coagulation times can be assessed by performing two tests that correspond to the two pathways of coagulation cascade activation (Figure 7). It is important to study both the extrinsic and the intrinsic coagulation times, since nanomaterials can produce a specific effect in only one of the pathways, which thus might be underestimated if only one is studied.

The prothrombin time (PT) test measures the activation of coagulation by the extrinsic or tissue factor. For the assessment of the coagulation time, once a sample is placed into the coagulometer, phospholipid calcium thromboplastin is added and the coagulation time is automatically assessed. Normal values for healthy humans are in the range between 12 and 15 seconds. 

The activated partial thromboplastin time (APTT) test consists of the measure of the activation of the contact or intrinsic coagulation pathway. To calculate this parameter, cephalin, a negatively charged phospholipid acting as a contact activator, is added to the sample previously placed in the coagulometer. The mixture is incubated for two minutes, followed by the addition of calcium chloride to activate the clot formation and measurement of the coagulation time. For this experiment, the normal values for healthy humans are between 25–35 s [40].

To perform the tests, it is important to obtain blood (human blood if possible) of high quality and maintain it in a non-coagulation environment. In addition, the use of fresh blood (stored at 4 ºC for a maximum of 2–3 days containing anticoagulant factors) is strongly recommended, since blood is damaged over time. As indicated above for other techniques, this technique is based in the use of a biological material, the blood, so variations between individuals, and even more with diseased individuals, are expected. Therefore, using blood samples from different individuals is highly recommended.

It must be taken into account that one of the key proteins of the coagulation cascade is fibrinogen (Figure 7). Therefore, nanomaterial interactions with this protein could influence the coagulation cascade [40]. For this reason, it is recommended that fibrinogen aggregation, if alterations on the coagulation cascade are found, is studied using the techniques indicated above, such as nanosystem stability in the presence of fibrinogen and fibrinogen electrophoresis after incubation with nanosystems.

### 2.2. Techniques to Study Interaction of Nanomaterials with Cells

Apart from interactions with proteins, when nanomaterials enter the body they also come into contact with non-targeted cells. The interaction of nanomaterials with one type of cell might be desired when, for example, aiming to transfect genes; however, it might be also undesired when the interaction is with immune cells. In addition, it must be taken into account that the presence of the nanomaterial or the presence of its degradation products can produce cell toxicity. Techniques to study both cell interaction and cell toxicity are summarized below.

#### 2.2.1. Desired Interaction with Cell Surface: Nanomaterial Uptake

Nanomaterial uptake is a key point in further achieving pharmacological action of a drug or the transfection of gene material. The most common mechanism of nanoparticle uptake is by endocytosis using different pathways (e.g., caveolae or clathrin) that depend on the properties of the nanomaterial. Therefore, nanosystems must be carefully designed in order to target the desired receptor. In addition, receptors specific to cell type must also be used; therefore, the nanomaterials need to have been previously functionalized with an active target moiety.

It is important to remark on the use of fluorescent dyes to follow nanosystem uptake, labeling both the nanosystem and cell components. The colocalization of the nanosystem dye with the subcellular structure labeled means that the nanosystem penetrates the cell through this route. In contrast to the techniques used to study nanomaterial interaction with proteins, cells are required for these studies. As in all techniques that use cell cultures, the results will depend not only on the properties of the nanomaterial studied, but also on the cell type used. In most studies, immortalized cell lines are used, due to their ease of use, commercial availability, and immortality. However, the results using primary cell lines are more reliable, since they come directly from biological individuals and have suffered fewer modifications than immortalized cell lines. Therefore, when translating to in vivo studies, the results are expected to be more similar. However, the obtaining of primary cell lines can be difficult for non-specialized groups, as working with them requires well-trained researchers. In addition, obtaining the samples can be complex and sometimes cannot be achieved without consequences for the donor.

#### 2.2.2. Phagocytosis Assessment

Phagocytosis is a specific kind of nanomaterial uptake that can be only performed by the so-called phagocytic cells, macrophages being the most representative. In contrast with endocytosis, phagocytosis is not desirable in most cases, since it represents the first step in the elimination of the nanosystem by the reticuloendothelial system (RES). Many parameters of the nanomaterial influence the phagocytic rate. For example, the PEGylation of the nanomaterial, together with an elongated shape, are factors that decrease the phagocytosis [18,40]. As cell penetration, phagocytosis can be studied in vitro taking advantage of fluorescence microscopy, which is the most commonly used technique, although there are other techniques that can be used, such as electron microscopy.

#### 2.2.3. Toxicity Assessment Techniques

Colloidal nanomedicines always have to be designed using biocompatible and biodegradable materials, thus achieving a safe therapy. However, the properties of nanomaterials sometimes differ from those of the materials of which they are made, and physiological interactions of nanomaterials with biological components may differ from those of isolated materials [2,3]. For this reason, before starting any in vitro and further in vivo assays, the nanomaterial toxicity has to be studied in depth using various cell lines and diverse toxicity tests, since each measures different parameters of the cells and have different sensitivities [41]. These tests, in general, have the advantage of a facile, rapid, and economic performance; more importantly, the use of animals, which should only be used when in vitro alternatives are not available, is reduced [42]. However, it should be noted that some results obtained in vitro cannot be extrapolated to in vivo behavior [42]. In the following sections, the most commonly used tests to assess nanomaterial toxicity in vitro, namely hemolysis and cytotoxicity, are discussed.

##### Hemolysis Determination

Erythrocytes or red blood cells (RBCs) represent the most numerous cells in the blood (around 45%). They are responsible for oxygen transport to the tissues and the removal of carbon dioxide, also contributing to the acid–base blood balance. Therefore, nanomaterial effects on erythrocyte integrity are a widely used measure of their toxicity in the blood [20,40]. The term hemolysis refers to the potential of a substance to damage erythrocytes, which may lead to their loss. It occurs when hemoglobin is released from a compromised or ruptured erythrocyte plasma membrane. Not only is the erythrocyte hemolysis a dangerous process for individual survival, but the released hemoglobin also represents a toxic component in the blood. 

**Figure 8:**
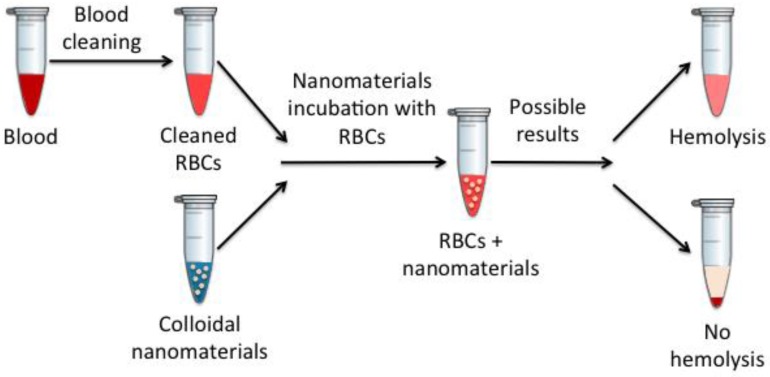
Schematic representation of the hemolysis assay. RBCs: red blood cells.

Hemolysis is one of the most commonly used techniques to study the effects that injected materials produce have on the blood, since, in this case, these in vitro results usually correlate quite well with in vivo results [40]. To measure the produced hemolysis, spectroscopic measures are performed in nanomaterials incubated with erythrocytes at different times, as schematically described in Figure 8.

It is worth noting that nanomedicine interactions with erythrocytes are largely determined by their physicochemical characteristics: mainly size, surface charge, and shape [40].

##### Cytotoxicity Assessment

Colloidal nanosystem cytotoxicity is a key parameter that must be studied when initiating in vitro assays, before any preclinical studies. The cytotoxic character of a nanomaterial can be given not only by its components but also by its physicochemical properties. It is remarkable that, although a bulk material is biocompatible and biodegradable in the nanomaterial form, it could be toxic [3]. In addition, the compatibility of nanosystems with biological tissues depends also on the tissue type as well as on the assay performed, since different assays measure different parameters related with cell viability. For this reason, it is strongly recommended that colloidal nanomaterial toxicity is studied in different cell lines, performing various toxicity tests, which are described below [41,42]. Nowadays, there are many fluorescent dyes such as propidium iodide that can be useful in determining cell viability under confocal microscopy or by flow cytometry. However, they have not been included because they are beyond the scope of this review.

• The MTT Test

The 3-(4,5-dimethylthiazol-2-yl)-2,5-diphenyltetrazolium bromide (MTT) colorimetric assay is, by far, the most widely used cytotoxicity tests in vitro, together with the MTS assay, which is a modification of the MTT [43,44,45]. This test assesses cell viability by means of measurement of the mitochondrial cell activity [45,46]. Since mitochondrial dehydrogenase enzymes cleave the tetrazolium ring, tetrazolium salts (the MTT reagent) are quantified to perform this test. After their formation, they are dissolved in dymethylsulfoxide, and their concentration is quantified by measuring the absorbance. The higher the absorbance, the higher the number of MTT crystals formed and thus the higher the cell viability. Together with the neutral red assay (detailed below), they are the most sensitive cytotoxicity tests [19,41].

Although MTT has been widely used, it does not always yield reliable results. Since it measures the mitochondrial activity of cells, when working with very slow dividing cells or even in confluence conditions (e.g., to reproduce the blood-brain barrier (BBB), a body structure that does not divide in normal conditions), metabolic cell activity is very low, thus resulting in a limited formation of crystals and resulting in a low absorbance that can barely be quantified. Therefore, in this case, MTT is not recommended.

• Lactate Dehydrogenase Assay (LDH) Assays

The lactate dehydrogenase assay (LDH assay) measures LDH activity in extracellular media, which gives an indication of the rupture of the cell membrane [40,46,47]. It was first developed to test the cytotoxicity in neuronal cells [48]. Specifically, this test measures the production of lactate and NAD+ from pyruvate and NADH, a reaction that only takes place in the presence of LDH enzyme, by measuring the absorbance of NADH [41,48]. Therefore, only in the presence of LDH, the absorbance should decrease due to the oxidation of NADH [48]. Since it is a measure of the extracellular LDH, the higher the LDH activity, the more damaged the cell membrane is and thus the lower the cell viability [19].

• Neutral Red

The neutral red assay is another spectrophotometric test to study nanomaterial cytotoxicity in cell cultures. It is based in the incubation of cell cultures with neutral red (toluylene red), which is internalized by live cells and accumulated in lysosomes. If the cell membrane is damaged, its uptake is decreased, and it can also leak out. Therefore, the higher the neutral red uptake, the higher the cell viability [19,41].

• Trypan Blue

The trypan blue assay is based in a principle similar to the neutral red assay. In this case, however, trypan blue, as a diazo dye, is only permeable to compromised cell membranes; therefore, only dead cells are stained in blue. The amount of cell death is quantified by optical microscopy observations and calculation of viability percentage [19]. This assay can also be performed with other similar dyes, such as that of the Evans blue assay.

Summarizing, a combination of at least two techniques is recommended when performing a safety profile of nanomaterials in vitro to have full and reliable results*.* Although only the MTT (or the alternative MTS) test is used in the vast majority of studies, as mentioned above, there are some specific cases in which it does not yield reliable results. For this reason, it is recommended that the MTT is performed in combination with another one. Among the other three described, trypan blue is used in most studies only for the qualitative assessment of the cells when passaging cultures. This is attributable to its easy and rapid performance, which ensures that the cells counted are alive. Neutral red is less used. Therefore, with the aim to compare with previous studies, it is recommended that the MTT is tested together with the LDH.

## 3. Conclusions

The design of nanosystems for personalized medicine requires a study of their interaction with biological components, since that will determine the nanomaterial’s half-life, fate, and biodistribution in the body. 

As has been described in this review, there are several techniques for studying nanomaterial interaction with proteins. Since most nanomaterials are designed to be administered through the intravenous route, it is important to apply a technique for the study of the general interaction with all blood proteins, as well as to specific techniques for the study of key proteins. Among blood proteins, it is recommended that the interactions with albumin are studied, because it is the most abundant protein in the blood; consequently, nanomaterials will rapidly find themselves surrounded by albumin. In addition, study of the interactions with C3 complement system protein are also encouraged, since this would enable a first indication of the reaction of the immune system through the designed nanomaterial. 

On the other hand, it is also important to study the interaction of nanosystems with cells. To achieve a personalized therapy, nanomaterials must be designed with a specific surface, targeting the desired receptor of a target cell. A first approximation of the study of nanomaterials uptake can be performed in vitro. However, there are also many non-desired interactions with other cells, which could produce cell and/or blood toxicity that must be known before in vivo studies. 

Most current studies focus on the therapeutic activity of nanomaterials, while studies of the safety profile are less common. Therefore, the translation of results to clinical studies is very difficult. To overcome this problem, a deep study of nanomaterial interaction with any kind of biological protein and/or cell is strongly recommended before translation to clinical trials so that novel personalized therapies can be establish.

## Figures and Tables

**Table 1:** Electrophoretic techniques useful for the detection of proteins in colloidal nanometric samples.

Technique	Characteristics that analyses	Advantages	Disadvantages	References
Sodium dodecyl sulfate–polyacrylamide gel electrophoresis (SDS-PAGE)	Proteins present in a sample separated as a function of their molecular weight	Simple and affordable	Requires preparative steps	[21,25,26,27,28]
Rocket immunoelectrophoresis and radial immunodifusion	Quantification of individual proteins Study of BSA interaction with nanoparticles	Simple, quick, and reproducible High accuracy Quantitative results Use of little equipment No preparative separation steps ^1^	Requires a calibration curve	[10,20,21,24,29,30,31]
2D immunoelectrophoresis	Quantification of individual proteins Study of the activation of the complement system (C3 protein)	Simple, quick, and reproducible Use of little equipment Quantitative results No preparative separation steps	Requires a calibration curve Difficult to extrapole to in vivo results Low sensitivity	[24,32,33,34]
